# NET Biomarkers in COVID-19 and Post-COVID Syndrome: a Comprehensive Analysis

**DOI:** 10.1007/s10875-026-01980-9

**Published:** 2026-02-02

**Authors:** Diana M. Monsalve, Laura Numpaque-Morales, Manuel Rojas, Yeny Acosta-Ampudia, Carolina Ramírez-Santana

**Affiliations:** 1 Center for Autoimmune Diseases Research (CREA), School of Medicine and Health Sciences, Universidad del Rosario, Carrera 24 No. 63-C-69, 111221 Bogotá, Colombia; 2 Estudiante Programa de Bacteriología y Laboratorio Clínico. Facultad de Ciencias de La Salud, Universidad Colegio Mayor de Cundinamarca (UCMC), Bogotá, Colombia; 3 Division of RheumatologyAllergy and Clinical Immunology, University of California, Davis, CA 95616 USA

**Keywords:** NETosis, Extracellular traps, Neutrophil, SARS-CoV-2, COVID-19, Post-COVID syndrome, Inflammation

## Abstract

SARS-CoV-2 triggers immune responses, including neutrophil activation and neutrophil extracellular trap (NET) release, contributing to COVID-19 severity. A significant proportion of patients experience persistent or new symptoms after recovery, referred to as post-COVID syndrome (PCS), which may be associated with NET release persistence. This study aimed to investigate whether NET-related biomarkers remain elevated after acute SARS-CoV-2 infection and to explore their association with PCS. We conducted an analytical descriptive cohort study including patients with acute COVID-19 (*n* = 35), PCS (*n* = 35), and a pre-pandemic healthy control group (PPC; *n* = 35). Serum samples from participants were used to measure NET biomarkers, including MPO-DNA complexes, elastase-DNA complexes, and MPO levels. The capacity to induce NETosis *in vitro* was evaluated by incubating neutrophils with participant serum. Additionally, soluble immune markers, including cytokines, anti-SARS-CoV-2 antibodies, and autoantibodies, were assessed. A NETosis-based model was developed using NET biomarkers to distinguish between acute COVID-19 or PCS patients and PPC individuals. Our findings revealed a significant increase in NET biomarkers during acute COVID-19 that persisted in PCS. This pattern was consistent with the *in vitro* study, where sera from PCS patients induced NET release. Additionally, NET biomarkers correlated with neutrophil counts, cytokines, autoantibodies, and inflammatory markers during acute COVID-19 and PCS. The multivariate model of NET biomarkers exhibited a high accuracy in distinguishing acute COVID-19 with an area under the curve (AUC) of 0.99 (CI = 0.98–1.00) and PCS patients with an AUC of 0.91 (CI = 0.84–0.99) from PPC. These findings suggest that persistent NETosis may contribute to PCS pathophysiology, highlighting NETs as potential biomarkers and therapeutic targets in post-viral syndromes.

**Supplementary Information:**

The online version contains supplementary material available at 10.1007/s10875-026-01980-9.

## Introduction

COVID-19 is an infectious disease caused by SARS-CoV-2, which triggered a global health crisis characterized by widespread morbidity and mortality. As of September 2025, more than seven million deaths have been officially reported worldwide [1]. The clinical manifestations of COVID-19 vary widely, ranging from asymptomatic cases to mild forms that primarily involve the upper respiratory tract to severe forms, including pneumonia, multi-organ failure, and, in some cases, death [2]. During the acute phase of COVID-19, immunothrombosis and endothelial dysfunction have been identified, together with systemic inflammation characterized by elevated cytokine levels [3–5].

Post-acute sequelae, commonly referred to as post-COVID syndrome (PCS), have emerged as a significant public health concern. The World Health Organization defines PCS as the continuation or appearance of new or persistent symptoms lasting at least two months with no alternative explanation [6]. The development of PCS has been associated with several factors, including female sex, older age, higher body mass index (BMI), smoking, preexisting comorbidities, and previous hospitalization or intensive care unit (ICU) admission [7]. PCS manifests as a multisystemic disorder characterized by pulmonary, musculoskeletal, digestive, and neurological manifestations, among others [8]. The severity and duration of these symptoms vary considerably among individuals, posing significant challenges for diagnosis and clinical management [9, 10].

The pathogenesis of PCS remains under research, with several hypotheses proposed. One prevailing theory suggests a persistent chronic inflammatory response involving both innate and adaptive immunity [8, 11, 12]. Neutrophils, as early responders, employ defense mechanisms such as phagocytosis, degranulation, and the formation of neutrophil extracellular traps (NETs) to neutralize pathogens. NETs are web-like structures composed of extracellular DNA, histones, and granule-derived enzymes such as myeloperoxidase (MPO) and elastase [13]. However, when NET formation is not properly regulated, these structures can exacerbate inflammation, tissue damage, and thrombosis [14]. In the context of SARS-CoV-2 infection, elevated neutrophil counts in the blood have been identified as early markers of disease severity, associated with poor respiratory outcomes and worse clinical prognosis. An exaggerated neutrophil response, specifically characterized by the overproduction of NETs, has been implicated in the pathogenesis of acute COVID-19 [2, 15]. More recently, sustained NET overproduction and accumulation have been proposed to contribute to the pathophysiology of PCS, promoting endothelial dysfunction, disseminated intravascular coagulation, and chronic systemic inflammation [16–18].

This study aimed to investigate the role of NETs in PCS. We evaluated several NET-associated biomarkers (i.e., MPO-DNA complexes, elastase-DNA complexes, and MPO levels) in the serum of patients with acute COVID-19, assessed their persistence during PCS, and explored their relationship with clinical and biological variables in both phases of the disease, while comparing them with unexposed individuals (i.e., PPC). Additionally, we evaluated the capacity of patient serum to induce NETosis *in vitro* using neutrophils from healthy donors.

## Materials and Methods

### Study Design

An analytical descriptive cohort study was conducted, comprising three groups: acute COVID-19 (*n* = 35), PCS (*n* = 35), and pre-pandemic healthy group (PPC; *n* = 35). The sample size was estimated based on previously published data reporting increased release of NETs in patients with COVID-19, quantified by fluorescence intensity [2]. Using those data as reference and assuming a moderate effect size, an alpha level of 0.05, a statistical power of 80%, and an expected 20% attrition rate, we applied the formula for mean difference as previously described [19], yielding a final sample size of 35 subjects per group. In previous studies conducted by the Center for Autoimmune Diseases Research (CREA), we included patients with acute COVID-19 (between July and August 2020) who were subsequently followed during the post-COVID phase (between March and May 2021) from Clínica del Occidente in Bogotá, Colombia [8, 20, 21].

Eligible participants were adults (≥ 18 years) with a confirmed SARS-CoV-2 infection by RT-PCR, hospitalized due to COVID-19, with a Sequential Organ Failure Assessment score < 6 at the time of recruitment, and classified as severe cases according to the Pneumonia Diagnosis and Treatment Scheme for Novel Coronavirus Infection (Trial Version 7). Patients were excluded if they were pregnant or breastfeeding, had a history of allergic reactions to transfusions, were critically ill and admitted to the ICU, had undergone surgery within the previous 30 days, were receiving active cancer treatment, had HIV infection with viral failure, presented another confirmed infection explaining the clinical manifestations, or had end-stage kidney disease, Child–Pugh C liver cirrhosis, high-output cardiac disease, autoimmune diseases, or immunoglobulin A nephropathy [20].

Thirty-five individuals with confirmed PCS and available serum samples from the acute phase were included in the study. These patients voluntarily attended the post-COVID unit. Participants with a history of SARS-CoV-2 vaccination, preexisting autoimmune diseases prior to acute infection, or without laboratory-confirmed COVID-19 were excluded [8]. For the PPC group, individuals were selected based on the absence of autoimmune diseases and no prior exposure to SARS-CoV-2. Additionally, neutrophil purification assays were performed using samples from five healthy post-pandemic volunteers. All participants provided informed consent and were notified about the Colombian Data Protection Law (Law 1581 of 2012). The study design received approval by the Institutional Review Board at Universidad del Rosario.

### Human Neutrophil Purification

Whole blood from healthy donors was collected into EDTA tubes. Leukocytes were isolated by dextran sedimentation of the red blood cell (RBC) layer. The leukocyte-rich upper phase was layered onto Ficoll-Hypaque (10771, Sigma-Aldrich) and centrifuged. The resulting pellet, containing RBCs and polymorphonuclear cells (PMNs), was treated with ammonium-chloride-potassium lysing buffer. PMNs were resuspended in complete RPMI-1640 medium (11875093, Gibco) with 10% FBS (s18b, Biowest). Neutrophil count and viability were assessed by trypan blue exclusion, and purity (> 95%) was confirmed by nuclear morphology (see Supplementary Methods).

### In-House Control NETs

To minimize inter-assay variability and address the lack of an international standard for measuring NET complexes, a stock solution of NET standard, defined as 100% NET, was prepared and used to generate a standard curve as described by Guy et al. [22]. The purified neutrophils were resuspended in complete RPMI-1640 medium and seeded at a density of 2.5 × 10^5^ cells per well. The cells were then stimulated with 100 nM phorbol 12-myristate 13-acetate (PMA; P8139, Sigma-Aldrich) as a positive control or left unstimulated as a negative control for 4 h at 37 °C in a 5% CO₂ atmosphere. Supernatants from stimulated and unstimulated neutrophils obtained from five independent donors were then pooled to prepare the in-house NET standard used for subsequent ELISA analyses (see Supplementary Methods).

### MPO-DNA Complexes

MPO-DNA complexes in serum were quantified using a sandwich ELISA adapted from methods previously described by Kessenbrock et al. [23] and Zuo et al. [2]. High-binding 96-well plates (762071, Greiner Bio-One) were coated overnight at 4 °C with 1 μg/mL anti-MPO antibody (0400–0002, Bio-Rad) in PBS. After 17 h, the plates were washed with 0.05% Tween-20 in PBS and blocked with 5% BSA (A3912, Sigma-Aldrich) for 2 h at RT. The plates were washed and incubated with NET control samples prepared by a series of consecutive 1:2 dilutions, starting from a 1:5 dilution or with 10% serum samples for 90 min. After washing, peroxidase-conjugated anti-DNA antibody (11774425001, Roche-Cell Death Detection ELISAPLUS), diluted 1:100, was added and incubated for 90 min. Following additional washes, the reaction was developed using TMB-substrate (T2885, Sigma-Aldrich) and stopped after 5 min with 2N H_2_SO_4_. Absorbance was measured at 450 nm with a 655 nm reference correction wavelength. The levels of MPO-DNA complexes were reported as a percentage relative to the NET-standard (see Supplementary Methods).

### Elastase-DNA Complexes

Elastase-DNA complexes in serum were measured using a modified PMN Elastase Human ELISA Kit (ab119553, Abcam), following the manufacturer’s instructions with a modification in the conjugate used for complex detection. Briefly, 96-well plates pre-coated with anti-PMN elastase antibody were incubated with NET control samples or serum samples diluted 1:10. After washing, a DNA-POD conjugated antibody, diluted 1:100 (from the Roche Cell Death Detection ELISAPLUS kit), was added and incubated. Plates were then washed and TMB substrate was added for color development and incubated for 20 min. The reaction was stopped by adding a stop solution and absorbance was measured at 450 nm with a reference wavelength correction at 655 nm (see Supplementary Methods).

### MPO

The MPO concentration in human serum was quantified using a commercial MPO ELISA kit (HK 324, Hycult Biotech), following the manufacturer’s instructions. Results were expressed in ng/mL.

### NETosis Induction by Serum Samples

Neutrophils (2.5 × 10^5^ cells per well) were seeded onto Poly-L-lysine-coated coverslips and incubated for 4 h at 37 °C under three conditions: unstimulated, stimulated with 100 nM PMA (positive control), or incubated with 10% serum. Cells were then fixed with 4% paraformaldehyde (150146, MP Biomedicals), permeabilized with 0.5% Triton X-100 (G5516, Sigma-Aldrich), and blocked with 5% BSA. To visualize NETs, cells were incubated overnight at 4 °C with an anti-MPO antibody (Ab9535, Abcam; 1:30), followed by a 2-h incubation at RT with an anti-DNA/Histone H1 antibody (Mab3864, Sigma-Aldrich; 1:100). After washing, cells were incubated with Alexa Fluor 488- and 546-conjugated secondary antibodies (A11001 and A11010, Thermo Fisher Scientific; 1:1000), counterstained with Hoechst 33342 (H3570, Thermo Fisher Scientific), and mounted using 70% glycerol. Images were acquired using a Leica DMi8 microscope and processed with Leica Application Suite X (LAS-X) software. The sample size for *in vitro* NETosis induction assays (*n* = 5 per group) was selected using weighted random sampling from each study group based on feasibility and sample availability.

### Cytokines

Cytokine levels, including IL-1β, IL-2, IL-4, IL-5, IL-6, IL-7, IL-8, IL-9, IL-10, IL-12p70, IL-13, IL-17A, TNF-α, G-CSF, GM-CSF, RANTES, MCP-1, IP-10, IFN-γ, and IFN-α, were measured using a Cytometric Bead Array kit (BD Biosciences™), according to the manufacturer’s instructions. Cytokine quantification was performed in samples from 11 acute COVID-19 patients, 24 PCS, and 14 PPC, except for IL-8, which was assessed in all samples. The analysis was performed using a FACSCanto II™ flow cytometer (BD Biosciences™), as previously described [24].

### IgG/IgA anti-SARS-CoV-2 Antibodies

Serological detection of human IgG and IgA antibodies against the SARS-CoV-2 S1 structural protein was performed on all samples using a Euroimmun ELISA kit (Euroimmun, Lübeck, Germany) following the manufacturer's instructions. Serum samples were diluted 1:100, and results were interpreted based on the ratio values: < 0.8 was considered negative, ≥ 0.8 to < 1.1 borderline, and ≥ 1.1 positive.

### Autoantibodies

A comprehensive panel of autoantibodies was analyzed, including IgM rheumatoid factor (RF), IgG anti-cyclic citrullinated peptide third-generation (CCP3), IgM and IgG anti-cardiolipin antibodies (ACAs), IgM and IgG anti-β2 glycoprotein-1 (β2GP1) antibodies, IgG anti-double-stranded DNA (dsDNA) antibodies, IgG anti-thyroglobulin (Tg) antibodies, and anti-thyroid peroxidase (TPO) antibodies. Quantification was performed using enzyme-linked immunosorbent assay (ELISA; INNOVA, Barcelona, Spain). The analysis included 11 COVID-19 patients, 11 PCS, and 29 PPC. Notably, all PCS patients were tested for RF and CCP3. The final Spearman correlation analysis included results from a proteomic microarray evaluating 116 IgG antibodies targeting self-antigens, SARS-CoV-2 antigens, and other common viral antigens in all PCS patients [25].

### Statistical Analysis

In the univariate analysis, categorical variables were analyzed using frequencies, and quantitative continuous variables were expressed as the mean and standard deviation (SD) or the median and interquartile range (IQR). No variable underwent statistical transformation or normalization. Given the longitudinal design of our study, paired t-tests were used to compare acute and PCS phases, while ANOVA with Dunnett’s post hoc test was applied for comparisons with the PPC group and sex-based subgroup analyses. Spearman correlation was used to explore associations between NETosis biomarkers in acute COVID-19 and PCS, with clinical laboratories, cytokines, SARS-CoV-2 antibodies, and autoantibodies, with significant correlations visualized using network analysis (tidygraph package). Finally, the receiver operating characteristic (ROC) curve and the area under the curve (AUC) were computed (pROC package) to assess the performance of MPO-DNA, elastase-DNA, and MPO in distinguishing acute COVID-19 and PCS from PPC, using a 50% cutoff. Sensitivity and specificity were calculated using the epiR package. In addition, we evaluated the performance when combining the three biomarkers. A *P* value of < 0.05 was set as significant. Analyses were performed using R version 4.2.1 and GraphPad version 10.0.2.

## Results

### Clinical Characteristics of Acute COVID-19 Patients

Patients with acute severe COVID-19 were included and evaluated as previously described [20]. Most of the patients were male (62.9%), with a median age of 50 years (IQR 41–56). The median time from symptom onset to inclusion was 9 days (IQR 7–11). All patients were hospitalized, and 17.2% were admitted to ICU. The most common symptoms at admission during the acute phase of COVID-19 reported by > 80% of patients were fatigue and malaise, dry cough, fever, dyspnea, myalgia, and arthralgia. The most prevalent comorbidities were acid peptic disease (40%), obesity (34.3%) and dyslipidemia (34.3%). Clinical and laboratory characteristics of these patients during the acute phase are detailed in Table S1. Compared with patients with COVID-19, PPC had a higher proportion of females (71.4%, *p* = 0.004) and were younger (median age of 40 years, *p*  < 0.006).

### Clinical Characteristics of PCS Patients

The same cohort of patients was re-evaluated during the post-COVID phase [8, 21], with a median post-infection interval of 217 days (IQR 186–238). None were vaccinated at the time of the study. The majority presented with persistent or new symptoms affecting multiple systems, predominantly musculoskeletal (74.3%), neurological (54.3%), and pulmonary (45.7%) manifestations. Scores reflecting residual symptom burden indicated mild to moderate dysfunction: the median Zung self-rating depression score was 32 (IQR 28.5–38.5), and the median COMPASS-31 score, evaluating autonomic symptoms, was 9 (IQR 4.5–14.5). Overall, these findings reveal the persistence of multisystemic manifestations and support the chronic inflammatory features characteristic of PCS in this cohort (Table S1).

### Assessment of NETs in the Sera of COVID-19 and PCS Patients

**Fig. 1:**
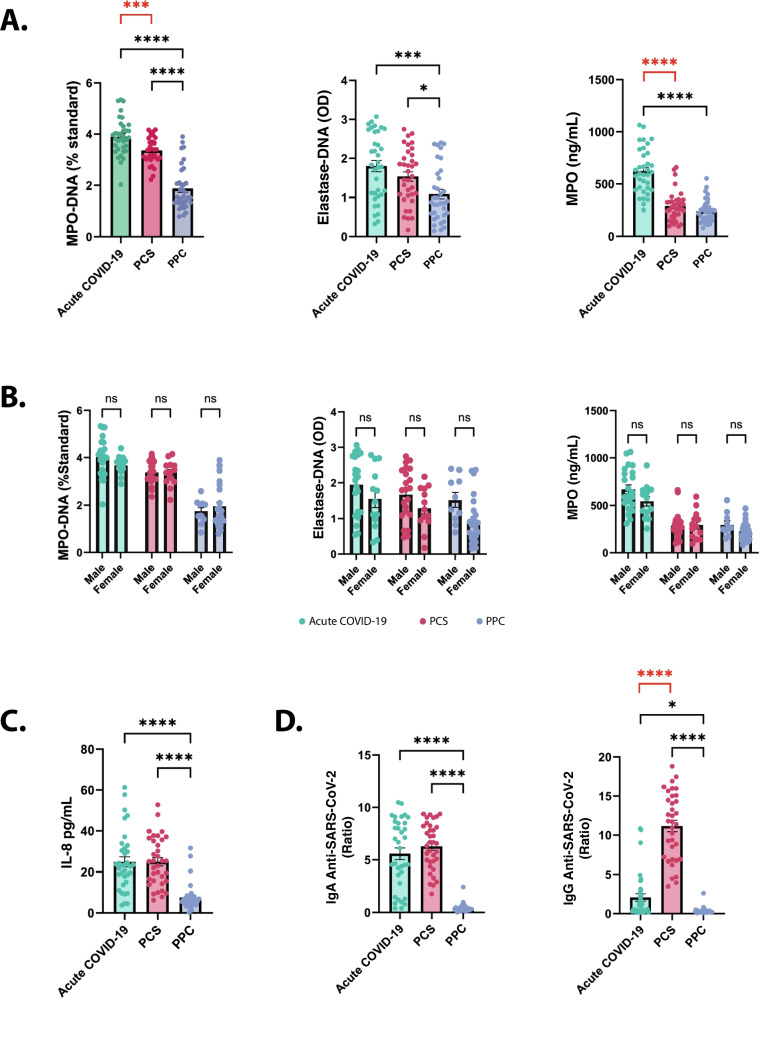
NETs biomarkers and soluble immune markers in sera of acute COVID-19, PCS and PPC. Serum samples from acute COVID-19 (*n* = 35), PCS (*n* = 35) and PPC (*n* = 35) were evaluated for **A** MPO-DNA complexes (% standard), Elastase-DNA complexes (OD), and MPO levels (ng/mL). **B** NET biomarker levels stratified by gender. **C** IL-8 levels and **D** IgA anti-SARS-CoV-2, and IgG anti-SARS-CoV-2 antibodies. Comparisons between acute COVID-19 or PCS samples and PPC were performed using ANOVA and adjusted for multiple comparisons by Dunnett-Test (significant differences indicated in black). Pairwise comparisons between acute COVID-19 and PCS groups were conducted using a paired t-test (significant differences indicated in red). *P < 0.1, **P < 0.01, ***P < 0.001, ****P < 0.0001. MPO: Myeloperoxidase, OD: Optical density, PCS: Post-COVID syndrome; PPC: Pre-pandemic healthy controls

To evaluate the presence of NETs in patients with acute COVID-19 or PCS, serum biomarkers associated with NETs were measured and compared with PPC. In the acute COVID-19 group, serum levels of MPO-DNA and elastase-DNA complexes were significantly elevated compared with PPC (adjusted *p* < 0.0001 and *p* = 0.0003, respectively). MPO concentrations were also markedly increased in this group (adjusted *p* < 0.0001). In the PCS group, these NET-associated biomarkers remained significantly elevated relative to PPC (adjusted *p* < 0.0001 for MPO-DNA and *p* = 0.0280 for elastase-DNA complexes), indicating the persistence of NETs long after the acute infection (Fig. 1A). When comparing these NET markers between the acute and PCS phases, the acute COVID-19 group displayed significantly higher levels of MPO-DNA (*p* = 0.0006) and MPO (*p* < 0.0001). However, no significant differences were observed for elastase-DNA complex levels (Fig. 1A).

Given the potential role of female sex hormones in promoting NETosis [26], we evaluated the impact of gender on NET-related biomarkers. However, no significant associations were found between gender and levels of MPO-DNA complexes, elastase-DNA complexes, or MPO in patients with acute COVID-19, PCS, or PPC (Fig. 1B).

### Assessment of Soluble Immune Markers in the Sera of COVID-19 and PCS Patients

Additionally, we analyzed a panel of cytokines, along with the presence of antibodies and autoantibodies, which could be implicated in triggering NETosis [27]. All assessed cytokines were significantly elevated in acute COVID-19 and PCS patients compared with PPC (Table 1). IL-8, a key neutrophil chemoattractant and inducer of NETosis, showed significantly higher levels in patients with acute COVID-19 (adjusted *p* < 0.0001) and PCS (adjusted *p* < 0.0001) compared with PPC (Fig. 1C). When comparing the acute and PCS phases, IL-6, IL-7, IL-10, and IP-10 were notably more elevated in acute COVID-19, whereas IL-2, IL-4, IL-1β, IL-9, GM-CSF, RANTES, and IFN-γ were more pronounced in PCS. The levels of other cytokines remained similar between both groups (Table 1).

**Table 1:** Soluble immune markers

Variable	COVID-19 (*n* = 35)	PCS (*n* = 35)	PPC (*n* = 35)	*P* value acute COVID-19 Vs PCS	*P* value all groups
Cytokines pg/mL Median (IQR)*					
IL-1β	4.46 (2.62—8.66)	13.89 (12.36—16.17)	0.00 (0.00—0.00)	0.040	< 1e-04
IL-2	10.26 (6.59—11.67)	11.93 (10.37—14.64)	0.00 (0.00—0.00)	0.019	< 1e-04
IL-4	0.00 (0.00—2.59)	11.39 (9.83—14.46)	0.00 (0.00—0.00)	< 0.001	< 1e-04
IL-5	1.69 (1.41—2.31)	2.86 (2.49—3.68)	0.00 (0.00—0.00)	0.568	< 1e-04
IL-6	119.03 (30.81—184.66)	21.36 (18.88—25.27)	0.00 (0.00—0.00)	< 0.001	< 1e-04
IL-7	29.02 (10.10—42.67)	13.30 (12.45—14.99)	0.00 (0.00—0.00)	0.043	< 1e-04
IL-8	23.06 (15.10—31.57)	25.00 (15.55—35.60)	5.86 (4.24—7.71)	0.991	< 1e-04
IL-9	3.99 (2.58—8.27)	20.80 (19.13—24.35)	0.00 (0.00—0.00)	< 0.001	< 1e-04
IL-10	13.12 (9.50 -15.99)	8.45 (7.44—9.84)	0.00 (0.00—0.00)	0.045	< 1e-04
IL-12p70	9.41 (2.68—11.60)	16.977 (14.22—23.27)	0.00 (0.00—0.00)	0.910	< 1e-04
IL-13	2.91 (1.14—3.81)	15.56 (13.71—17.73)	0.00 (0.00—0.00)	0.461	< 1e-04
IL-17A	24.65 (8.03—30.86)	55.34 (48.38—63.01)	0.00 (0.00—0.00)	0.434	< 1e-04
IFN-α	20.69 (13.25—30.53)	18.27 (16.37—24.16)	0.00 (0.00—0.00)	0.302	< 1e-04
TNF-α	2.91 (1.70—22.80)	21.81 (20.47—24.27)	0.00 (0.00—0.00)	0.591	< 1e-04
G-CSF	18.41 (6.22—52.87)	24.66 (22.84—28.71)	0.00 (0.00—0.00)	0.609	< 1e-04
GM-CSF	5.06 (3.55—6.86)	10.17 (8.09—15.75)	0.00 (0.00—0.00)	0.006	< 1e-04
RANTES	11565.40 (10117.23—14393.65)	43338.96 (37182.75—52169.72)	21496.43 (14393.08—22844.86)	< 0.001	< 1e-04
MCP-1	186.53 (106.05—289.30)	105.36 (50.56—252.15)	53.29 (30.86—82.40)	0.151	0.0055
IFN-γ	9.17 (8.67—12.20)	18.272 (16.37—24.16)	0.00 (0.00—0.00)	0.005	< 1e-04
IP-10	5277.77 (2341.01—8475.48)	32.61(27.20—40.81)	-	< 0.001	-
Anti-SARS-CoV-2 antibodies					
IgA positivity (%)	30 (85.7%)	35 (100.0%)	1 (2.85%)	0.020	0.0005
IgA ratio—median (IQR)	6.107 (3.16—8.50)	6.561 (4.85—8.16)	0.330 (0.25, 0.50)	0.290	< 1e-04
IgG positivity (%)	17 (48.6%)	35 (100.0%)	1 (2.85%)	< 0.001	0.0005
IgG ratio—median (IQR)	0.906 (0.40—2.52)	11.721 (7.08—14.75)	0.273 (0.240—0.34)	< 0.001	< 1e-04
Autoantibodies positivity (%)^†^					
RF	3/11 (27.3%)	14/35 (40.0%)	1/29 (3.4%)	0.446	0.0015
CCP3	0/11 (0.0%)	1/35 (2.9%)	0/29 (0.0%)	0.571	1.0000
dsDNA	0/11 (0.0%)	1/11 (9.1%)	0/29 (0.0%)	0.306	0.4453
TPO	5/11 (45.5%)	3/11 (27.3%)	3/29 (10.3%)	0.375	0.0510
Tg	0/11 (0.0%)	0/11 (0.0%)	0/29 (0.0%)	1.000	-
IgM β2GP1	0/11 (0.0%)	1/11 (9.1%)	1/29 (3.4%)	0.306	0.6782
IgG β2GP1	0/11 (0.0%)	0/11 (0.0%)	0/29 (0.0%)	1.000	-
IgM ACA	2/11 (18.2%)	0/11 (0.0%)	0/29 (0.0%)	0.138	0.1524
IgG ACA	0/11 (0.0%)	1/11 (9.1%)	0/29 (0.0%)	0.306	0.5102

^*^Cytokine quantification was performed in samples from 11 acute COVID-19 patients, 24 PCS and 14 PPC, with the exception of IL-8, which was assessed in all samples. ^†^Antibodies positivity was assessed in 11 acute COVID-19 patients, 11 PCS and 29 PPC. Only RF and CCP3 were tested for all PCS patients. Pairwise comparisons between acute COVID-19 and PCS groups were conducted using a paired T-test. *ACA* Anti-cardiolipin antibodies; *β2GP1* β2 glycoprotein-1; *CCP3* Cyclic citrullinated peptide third-generation; *dsDNA* Double-stranded DNA; *IQR* Interquartile range; *INR* International normalized ratio; *PCS* Post-COVID syndrome; *PPC* Pre-pandemic healthy controls; *RF* Rheumatoid factor; *SD* Standard deviation; *Tg* Thyroglobulin; *TPO* Thyroid peroxidase

As expected, anti-SARS-CoV-2 antibody levels were significantly elevated in both acute COVID-19 and PCS patients compared with PPC (Table 1). IgA levels were markedly increased in both groups (adjusted *p* < 0.0001; Fig. 1D), whereas IgG levels were significantly higher in PCS patients (adjusted *p* < 0.0001) and only modestly elevated in acute cases (adjusted *p* = 0.0352).

Although overt autoimmunity is not present in all COVID-19 patients, we assessed the presence of latent autoimmunity. The frequencies of autoantibodies in acute COVID-19, PCS, and PPC are shown in Table 1. Among them, RF was the only autoantibody significantly more frequent in acute COVID-19 and PCS patients compared with PPC (*p* = 0.0015). However, no significant differences in autoantibody prevalence were observed between acute COVID-19 and PCS (Table 1).

### Induction of NETosis in Healthy Neutrophils by Patient Sera *in vitro*

**Fig. 2:**
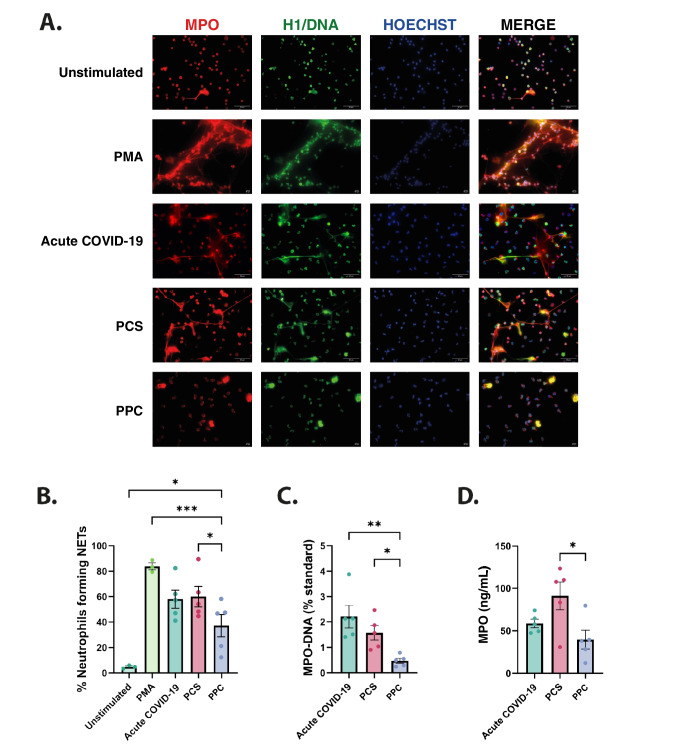
NETosis induction in healthy neutrophils. Serum samples from acute COVID-19 (*n* = 5), PCS and PPC (*n* = 5) were evaluated for their capacity to induce NETs release in neutrophils isolated from healthy controls. Samples were selected from the total sample using a weighted random sampling method to ensure a representative distribution across the three evaluated groups. **A** Indirect immunofluorescence staining was performed using antibodies against MPO (red), anti-DNA/Histone H1 (green), and Hoechst 33342 (blue) to visualize NETs. Representative images of unstimulated neutrophils, neutrophils stimulated with 100 nM PMA (negative and positive controls, respectively), and neutrophils stimulated with serum from acute COVID-19, PCS and PPC are shown. **B** NET formation was quantified using ImageJ. The percentage of NETs was determined by dividing the number of NET events observed in the MPO-DNA/H1 fluorescence channels by the total number of events in the Hoechst channel, then multiplying by 100. **C** MPO-DNA complexes and **D** MPO levels were detected in the supernatant of stimulated neutrophils. Data from acute COVID-19, PCS and PPC groups were compared using ANOVA and adjusted for multiple comparisons by Dunnett-Test (*P < 0.1, **P < 0.01, ***P < 0.001, ****P < 0.0001). H1: Histone 1, MPO: Myeloperoxidase, PMA: Phorbol myristate acetate, PCS: Post-COVID syndrome; PPC: Pre-pandemic healthy controls

Given the proinflammatory nature of COVID-19 and its association with NETosis, we assessed the NET-inducing capacity of serum from acute COVID-19 and PCS patients using neutrophils from healthy donors. Sera from the acute COVID-19 group showed a trend toward increased NET formation compared with PPC, although this difference did not reach statistical significance. In contrast, sera from the PCS group significantly induced NET formation compared with PPC (*p* = 0.0334) (Fig. 2A, B). These findings indicate that PCS serum retains the NET-inducing potential observed during acute infection. Additionally, the presence of NETs was verified by ELISA quantification of MPO-DNA complexes and MPO levels in the supernatants. MPO-DNA complex levels were significantly higher in acute COVID-19 (*p* = 0.0035) and PCS (*p* = 0.0498) compared with PPC. In contrast, MPO levels were significantly increased only in PCS (*p* = 0.0173). No significant differences were found between acute COVID-19 and PCS (Fig. 2C and D).

### Correlation of NETs Markers with Clinical and Inflammatory Biomarkers in COVID-19 and PCS

**Fig. 3:**
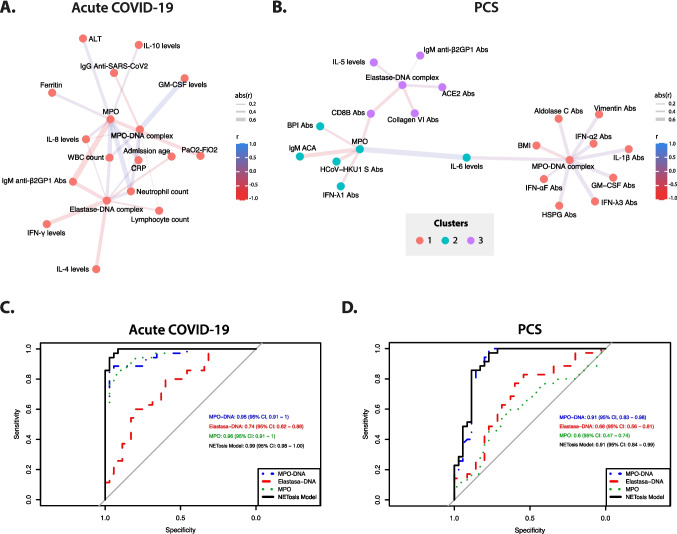
Correlation network between NET markers and clinical and inflammatory variables and NETosis model. **A** and** B** Spearman correlation network of NET-associated biomarkers and clinical and inflammatory variables for acute COVID-19 and PCS. Nodes represent the measured variables, and edges (lines) indicate inferred associations. Positive correlations are depicted with solid blue edges, while negative correlations are shown in red. The width of each edge is proportional to the strength of the correlation. **C **and** D** ROC curve analysis was performed to evaluate the accuracy of NET markers in distinguishing acute COVID-19 or PCS patients from PPC. The predictive ability for acute COVID-19 or PCS occurrence was compared among MPO-DNA complexes, elastase-DNA complexes and MPO levels, both individually and in combination

Next, a correlation network was constructed to explore relationships among clinical and biological parameters in acute COVID-19. The network displays only the significant correlations (Fig. 3A and Table S2). Positive correlations were observed between MPO-DNA complexes and GM-CSF, neutrophil count, white blood cell (WBC) count, C-reactive protein (CRP) and IL-8. Elastase-DNA complexes correlated with neutrophil count, WBC count, CRP, and IL-8. MPO levels showed positive correlations with neutrophil count, WBC count, CRP, as well as with alanine transaminase (ALT), ferritin, IL-8 and IL-10. In contrast, negative correlations were observed between MPO-DNA levels and IgG anti-SARS-CoV-2 antibodies and weakly with lymphocyte count. Among NET biomarkers, elastase-DNA exhibited the highest number of negative correlations, including associations with IFN-γ, IL-4, IgM anti-β2GP1 antibodies, age of admission and lymphocyte count. Additionally, MPO levels were negatively correlated with IgM anti-β2GP1 antibodies and PaO2-FiO2.

Next, we explored the association between serum NET-associated marker levels, biological parameters -including a more extensive autoantibody repertoire analyzed via proteomic microarray (see Materials and Methods section)- and clinical variables in PCS (Fig. 3B and Table S3). The correlation network analysis revealed the segregation of three distinct clusters represented by NET biomarkers. In the first cluster MPO-DNA complexes showed a positive correlation with autoantibodies against IFNs, heparin sulfate proteoglycan (HSPG), aldolase C, vimentin, IL-1β, and GM-CSF, as well as with IL-6 levels, while exhibiting a negative correlation with BMI. In the second cluster, characterized by MPO levels, a positive correlation was observed only with IL-6 levels, while all other associations were negative. These included correlations with IgM ACA and antibodies against HCoV-HKU1 S, CD8B, IFN-λ1, and bactericidal/permeability-increasing protein (BPI). In addition, the third cluster represented by elastase-DNA complexes, showed a positive correlation with IL-5 levels, anti-ACE2 antibodies and, to a lesser extent, IgM β2GP1. Conversely, negative correlations were observed with antibodies against CD8B and collagen VI.

Additionally, we explored whether NET biomarker levels varied across the clinical subphenotypes of PCS previously defined by our group based on symptom severity [8], and found no significant differences among clusters, suggesting that NET levels are not associated with disease severity.

### NETosis Model

Additionally, a ROC curve analysis was performed to assess the performance of NETosis-associated biomarkers in distinguishing acute COVID-19 and PCS patients from PPC (Fig. 3C and D). In acute COVID-19, MPO levels exhibited the highest accuracy, with an AUC of 0.96 (confidence interval CI = 0.91–1.00) closely followed by MPO-DNA complexes (AUC = 0.95, CI = 0.91–1.00), and elastase-DNA complexes (AUC = 0.74, CI = 0.62–0.86). The performance of the three biomarkers in a single model achieved an AUC of 0.99 (CI = 0.98–1.00), with a sensitivity and specificity of 94.3% (CI = 80.8%–99.3%) for identifying acute COVID-19 when compared with PPC (Fig. 3C). For PCS, MPO-DNA complexes exhibited the highest predictive accuracy (AUC = 0.91, CI = 0.83–0.98), followed by elastase-DNA complexes (AUC = 0.68, CI = 0.56–0.81), and MPO levels (AUC = 0.6, CI = 0.47–0.74). The NETosis model for PCS patients achieved an AUC of 0.91 (CI = 0.84–0.99), with a sensitivity of 80.0% (CI = 63.1%–91.6%) and specificity of 88.6% (CI = 73.3%–96.8%) for patient classification (Fig. 3D).

## Discussion

Several studies have highlighted the pivotal involvement of NETs in the development of various metabolic, autoimmune, and autoinflammatory disorders, contributing to increased morbidity and mortality [14, 28, 29]. In COVID-19, the pathological processes, including the cytokine storm resulting from uncontrolled activation of the immune system and the elevated incidence of thrombotic events, are closely linked to both the functional activity and dysregulation of neutrophils [30]. Elevated NET levels in both peripheral blood and lung tissues of COVID-19 patients contribute to disease severity and poor clinical outcomes [2, 31, 32]. In our study, we detected NET-related complexes, such as MPO-DNA and elastase-DNA, which provide accurate measurements of NET fragment concentrations in serum. Notably, some measurements are reported as surrogate markers of NETs, based on the quantification of individual components like MPO or citH3. As expected, our results revealed elevated levels of NET-associated biomarkers in the sera of acute COVID-19 patients. Although these biomarkers decreased in the PCS, they did not return to levels observed in PPC. This persistent elevation of NET-associated biomarkers in the post-COVID phase likely contributes to the clinical manifestations observed in our studied population [16].

Although information remains limited, few studies have demonstrated the presence of NETs in PCS. In this regard, plasma NET marker levels were evaluated in 279 individuals with mild and severe COVID-19 and long COVID patients, showing that neutrophilic elastase, MPO, and cfDNA may persist up to six months or longer after COVID-19 infection [33]. Additionally, proteomic analysis of PCS patients identified a distinct subset characterized by inflammatory immune signatures, strongly indicative of increased neutrophilic activity, correlating with biomarkers of degranulation and NET formation [11]. Other studies have proposed a potential link between NETs and complications associated with PCS, drawing on observations made during the acute phase of COVID-19 [34–36]. These findings offer valuable insights into the involvement of NETs in PCS and their role in autoimmunity, as well as pulmonary, cardiovascular, and neurologic complications associated with the condition [17].

Among the soluble immune markers analyzed, all evaluated cytokines were increased during the acute and PCS phases compared with PPC, which may contribute to NET formation. Specifically, cytokines can induce NOX-dependent NETosis by triggering calcium ion release into the cytoplasm, activating the PKC/Raf/MEK/ERK signaling pathway [37]. In particular, IL-8 levels were significantly elevated in acute COVID-19 and remained high during the post-COVID phase without returning to baseline levels observed in PPC. IL-8 is a potent chemoattractant that plays a crucial role in neutrophil recruitment, activation, and NET formation by binding to its receptors, CXCR1 and CXCR2 [38, 39]. Interestingly, we identified two distinct cytokine profiles when comparing acute COVID-19 and PCS, highlighting that while chronic inflammation persists in PCS, it differs from the acute phase. Several studies have reported a sustained elevation of proinflammatory cytokines in PCS patients [40, 41]. Thus, the persistent elevation of IL-8 in PCS, which may contribute to sustained neutrophil activation and NETosis, along with the concurrent presence of other proinflammatory cytokines, likely amplifies this effect by maintaining a chronic inflammatory milieu [21, 42]. This environment fosters ongoing NETosis and may explain the presence of NET biomarkers detected in the sera of our patients. A study by Cheong et al. [43] investigated transcriptional and epigenetic changes in PBMCs from COVID-19 convalescent patients, identifying CD14 + monocytes as the subset with the most drastic changes. Notably, one cluster, M.SC3, remained abundant even 12 months post-infection. These post-COVID monocytes displayed up to a 100-fold increase in proinflammatory cytokine secretion and showed enhanced transcriptional activity associated with cytokine signaling and monocyte activation. These changes represent a form of “trained immunity” or innate immune memory, in which innate immune cells undergo epigenetic reprogramming following SARS-CoV-2 infection.

On the other hand, we observed a significant increase in IgA and IgG antibody levels in response to SARS-CoV-2 infection, with IgG anti-SARS-CoV-2 levels remaining elevated in PCS, as expected. IgA can enhance neutrophil activation by promoting NETosis through Fc-dependent effector functions, specifically via engagement of the FcαRI/CD89 receptor. Notably, this enhancement is not virus-specific and has been observed for influenza A virus, HIV, and SARS-CoV-2 spike-pseudovirus [44, 45]. In this context, immune complexes formed by SARS-CoV-2-specific IgA and IgG have been shown to induce NET formation [46, 47]. Our results indicate that NETosis is higher during acute COVID-19 than in PCS, likely due to an additive effect mediated by IgA anti-SARS-CoV-2 antibodies.

Although our patients did not exhibit significant differences in the frequency of the evaluated autoantibodies, except for RF, several studies have reported the presence of latent autoimmunity, including antinuclear antibodies, RF, anti-neutrophil cytoplasmic antibodies, ACA, and anti-β2GPI antibodies, as well as autoantibodies targeting cytokines, chemokines, complement components, and cell surface proteins, all of which have been associated with the severity of COVID-19 and PCS [25, 48, 49]. Autoantibodies can induce NETosis, and the subsequent release of NETs upon activation serves as a source of autoantigens, triggering autoantibody production [50]. Altogether, the soluble immune markers evaluated in our study may contribute to a persistent proinflammatory state, perpetuating immune dysregulation and tissue damage, and thereby further contributing to the pathophysiology of PCS [51].

NET formation induced by components in biological samples in COVID-19 has been evaluated in other studies demonstrating that incubation of neutrophils isolated from healthy adults with plasma or serum from COVID-19 patients induces significant NET release [2, 52, 53]. Building on these findings, we observed that sera from patients in the post-COVID phase retained the capacity to significantly induce NET formation in healthy neutrophils. Furthermore, these findings were confirmed by the measurement of MPO-DNA complexes and MPO levels. In line with our results, a study conducted in Israel reported that NETosis induction levels were significantly higher in patients with PCS compared with recovered patients, showing that NETosis induction levels may reflect the underlying pathophysiology of this syndrome due to previous and possibly still increased NETosis with immune, thrombosis and, organ damage [18]. When comparing MPO-DNA complexes in sera and supernatants of stimulated neutrophils, we observed a similar pattern in their levels across the evaluated groups. However, MPO levels differed between sample types, in sera, only the acute COVID-19 group exhibited significantly higher levels compared with PPC, reflecting systemic circulation and overall inflammatory responses.

Among the NET biomarkers evaluated in acute COVID-19, MPO-DNA and elastase-DNA complexes showed correlations with inflammatory markers, including WBC count, neutrophil count, GM-CSF, CRP, and IL-8. Similar observations were reported by Zuo et al. [2] and Ng et al. [54], where markers of NET formation were associated with WBC count, neutrophils, inflammatory cytokines, and CRP. Interestingly, the intricate interplay and cross-talk among proinflammatory cytokines such as IL-8, GM-CSF, IL-6, TNF-α, IFNs, and IL-1β not only activate neutrophils to produce NETs but also perpetuate a self-amplifying inflammatory cascade, which exacerbates inflammation, oxidative stress, and thrombotic events [37]. In addition, the acute-phase reactant CRP, a reflex of IL-6, produced in the liver, is a common indicator of both acute infection and inflammation, which correlates with neutrophil count and NET release in COVID-19 [2, 37]. Similarly, MPO levels correlate with these markers, as well as with IL-10, ferritin, and ALT, all of which are linked to the inflammatory process and are elevated in COVID-19. Specifically, IL-10 has been associated with severity and mortality in both acute or post-acute SARS-CoV-2 infection [55]. Notably, this cytokine can delay NET formation, likely as a mechanism to limit hyperinflammation [56, 57]. However, recent evidence suggests that early IL-10 induction may act as a negative feedback signal that later shifts toward a proinflammatory role, amplifying cytokine production and potentially exacerbating the cytokine storm and NETosis [58]. In addition, elevation of liver enzymes is common in acute COVID-19. For instance, ferritin can promote NET formation through mechanisms dependent on peptidylarginine deiminase 4, neutrophil elastase, and reactive oxygen species, thereby intensifying the inflammatory response [59].

In PCS, MPO-DNA complexes correlated with several autoantibodies (proteomic microarray) and with IL-6. New-onset autoantibodies against a wide range of antigens (cytokines, chemokines, cell surface and nuclear antigens, etc.) detected in acute COVID-19 can remain elevated for at least 12 months and are associated with clinical manifestations in PCS [25, 60]. Moreover, persistent inflammation driven by cytokines, including IL-6, may contribute to the development of autoimmunity in PCS. Although IL-6 levels were higher in acute COVID-19 than in PCS, this cytokine remains elevated in PCS compared with PPC, indicating a persistent inflammatory state. This inflammatory milieu, characterized by the presence of autoantibodies and cytokines, can further activate neutrophils, leading to increased NET production and fueling a self-perpetuating inflammatory loop [33, 61]. Thus, dysregulated humoral responses, combined with the sustained presence of NETs, play a key role in initiating and perpetuating autoimmunity [17]. Nevertheless, further studies are needed to determine whether this latent autoimmunity progresses to overt autoimmune diseases in the future. In addition, MPO levels and elastase-DNA complexes, although positively correlated with the proinflammatory cytokines, these markers were negatively correlated with several autoantibodies such as, anti-CD8B, anti-IFN, ACAs and anti-β2GP1. The reasons for this unexpected association remain unclear, underscoring the need for further investigation to elucidate the underlying mechanisms. In line with our previous phenotypic stratification of PCS [8], the present study found no significant variation in NET biomarkers across severity clusters. These findings suggest that NETs may serve as diagnostic rather than prognostic markers in PCS, although their persistence could potentially predispose to long-term immune dysregulation.

Lastly, we developed a model based on NETosis-associated biomarkers to distinguish patients with acute COVID-19 and PCS from PPC. Among these, MPO levels and MPO-DNA complexes exhibited the highest accuracy for acute COVID-19, while MPO-DNA complexes showed the highest accuracy for PCS compared with other markers. However, when all NETosis markers were combined, the model achieved the highest predictive accuracy for acute COVID-19 with an AUC of 0.99, whereas for PCS the model had a predictive accuracy similar to MPO-DNA complexes alone with an AUC of 0.91, demonstrating good sensitivity and specificity. Several studies employing ROC analysis have highlighted the clinical significance of quantifying NETosis biomarkers. These biomarkers demonstrate high performance in distinguishing between healthy individuals and those with disease, as well as in monitoring disease progression in contexts such as cancer, sepsis, and autoimmune disorders [62–65]. In COVID-19, the accuracy of these markers has also been evaluated, showing potential as tools for monitoring patients infected with SARS-CoV-2 [32, 66]. However, data on PCS remain scarce. One study assessed the diagnostic performance of neutrophilic elastase, MPO, and circulating DNA, reporting AUC-ROC values of 0.64, 0.82, and 0.93, respectively, all statistically higher compared with healthy individuals [33]. These findings suggest that developing an optimal test combining multiple NET biomarkers could have significant clinical utility in acute COVID-19 and PCS. However, further studies are needed, including a cohort of individuals with a history of SARS-CoV-2 infection who did not develop PCS, to better delineate the specificity of these biomarkers.

This study has several limitations related to its retrospective design. Because data and serum samples were collected at different phases, some variables could not be uniformly assessed across all participants or time points. Differences in sample availability, timing of collection, and completeness of clinical data may have introduced variability in certain analyses. The patients included in the acute and post-COVID phases were not matched for age and sex with the PPC, which may have introduced bias. Several clinical and inflammatory variables were not evaluated in the same manner for acute COVID-19 and PCS. For instance, the proteomic microarray for autoantibody detection was performed only in PCS. In the MPO-DNA and elastase-DNA ELISA assays, we did not employ a calibration curve to obtain quantifiable concentrations of these complexes, as no commercial calibrators or standardized reference materials are currently available. The sample size for NETosis induction using neutrophils from healthy controls was limited to only five samples per group. This may explain why no significant differences were observed between acute COVID-19 and PPC, despite acute COVID-19 and PCS displaying similar results. Additionally, the inclusion of a group of patients with a history of COVID-19 who did not develop PCS would allow a better assessment of the specificity and significance of NETosis biomarkers and the proposed model. Furthermore, the absence of standardized classification criteria and validated severity scales for PCS represents another limitation, as these would be essential to determine whether NETosis is associated with different PCS stages or severity levels.

In summary, this longitudinal retrospective cohort study assessed MPO-DNA, elastase-DNA, and MPO as NET-associated biomarkers and evaluated their correlation with biological and clinical parameters. The findings revealed a strong association between NETs and acute COVID-19, as well as their persistence in PCS. Uncontrolled NETosis activation triggered by SARS-CoV-2 infection may be perpetuated by a feedback loop driven by a proinflammatory milieu, leading to increased systemic NET production and the development of PCS. This study advances current understanding by providing longitudinal evidence that sustained NET activation acts as a mechanistic bridge between the acute inflammatory response and the chronic immune dysregulation characteristic of PCS. By integrating clinical, serological, and functional NET data, our findings expand existing knowledge on the immunopathogenesis of PCS and reinforce the role of neutrophils as key mediators in the transition from acute infection to long-term sequelae. NET-associated biomarkers show potential clinical utility in both the acute and post-acute phases. The development of specific commercial assays for detecting MPO-DNA and elastase-DNA complexes could enable their use as diagnostic and monitoring tools. Moreover, elucidating the contribution of persistent NET biomarkers to the pathophysiology of PCS is essential for establishing these structures as viable targets for personalized therapeutic intervention. Prospective studies are warranted to translate these findings into practical clinical applications, including prognosis evaluation, clinical decision-making, and therapeutic planning.

## Supplementary Information

Below is the link to the electronic supplementary material.

Supplementary file1 (DOCX 24 KB)

Supplementary file2 (DOCX 17 KB)

Supplementary file3 (DOCX 17 KB)

Supplementary file4 (DOCX 20 KB)

## Data Availability

The data can be accessed upon a reasonable request by contacting the corresponding author.
