# Supplementary material for: NET Biomarkers in COVID-19 and Post-COVID Syndrome: a Comprehensive Analysis
Source: J Clin Immunol. 2026 Feb 2;46(1):19. doi: 10.1007/s10875-026-01980-9 (PMC12909450; doi:10.1007/s10875-026-01980-9)
Supplement: Supplementary file 1 — Supplementary file1 (DOCX 24 KB) [file 10875_2026_1980_MOESM1_ESM.docx]

**Table S1.** General characteristics of patients in the acute and post-COVID phases.

| **Variable** | **Acute COVID-19 / PCS patients (n=35)** | **PPC (n=35)** | **P value** |
| --- | --- | --- | --- |
| **Gender**  Female  Male | 13 (37.1%)  22 (62.9%) | 25 (71.4%)  10 (28.6%) | 0.004 |
| **Age (Median – IQR)** | 50 (41 - 56) | 40 (34 - 51) | 0.006 |
| **BMI (Median – IQR)** | 29.05 (26.71 - 30.86) | 24.60 (22.24 – 27.34) | < 1e-04 |
| **Symptoms on admission (%)** |  |  |  |
| Fever | 31 (88.6%) | - | - |
| Hemoptysis | 3 (8.6%) | - | - |
| Dry cough | 32 (91.4%) | - | - |
| Cough sputum | 16 (45.7%) | - | - |
| Sore throat | 15 (42.9%) | - | - |
| Anosmia | 20 (57.1%) | - | - |
| Dysgeusia | 22 (62.9%) | - | - |
| Rhinorrhea | 19 (54.3%) | - | - |
| Wheezing | 11 (31.4%) | - | - |
| Chest pain | 21 (60.0%) | - | - |
| Dyspnea | 30 (85.7%) | - | - |
| Lower chest wall indrawing | 1 (2.9%) | - | - |
| Myalgia | 29 (82.9%) | - | - |
| Arthralgias | 29 (82.9%) | - | - |
| Fatigue and malaise | 33 (94.3%) | - | - |
| Inability to walk | 8 (22.9%) | - | - |
| Headache | 27 (77.1%) | - | - |
| Abdominal pain | 8 (22.9%) | - | - |
| Nausea/vomiting | 19 (54.3%) | - | - |
| Diarrhea | 19 (54.3%) | - | - |
| Conjunctivitis | 1 (2.9%) | - | - |
| **Comorbidities (%)** |  |  |  |
| Hypertension | 5 (14.3%) | - | - |
| Dyslipidemia | 12 (34.3%) | - | - |
| Asthma | 2 (5.7%) | - | - |
| Stroke | 1 (2.9%) | - | - |
| Acid peptic disease | 14 (40.0%) | - | - |
| Diabetes | 3 (8.8%) | - | - |
| Obesity | 12 (34.3%) | - | - |
| Hypothyroidism | 4 (11.4%) | - | - |
| Malnutrition | 2 (5.7%) | - | - |
| Current smoker | 1 (2.9%) | - | - |
| Former smoker | 15 (42.9%) | - | - |
| **Days from symptoms onset to inclusion (Mean - SD)** | 8.94 (2.85) | - | - |
| **Hospitalization (%)** | 35 (100.0%) | - | - |
| **Total days of hospitalization (Mean - SD)** | 9.86 (7.07) | - | - |
| **ICU admission (%)** | 5 (17.20%) | - | - |
| **4C Mortality score (Mean - SD)** | 5.98 (2.08) | - | - |
| **SOFA on inclusion (Median - IQR)** | 2 (2–2) | - | - |
| **PaO2-FiO2 on inclusion (Median - IQR)** | 239 (214.55 – 287.70) | - | - |
| **Pharmacological therapy (%)** |  | - | - |
| Corticosteroids | 28 (80.0%) | - | - |
| Antibiotics | 35 (100.0%) | - | - |
| ACE inhibitors | 1 (2.9%) | - | - |
| Heparin | 32 (91.4%) | - | - |
| ARB II | 3 (8.6%) | - | - |
| **Admission laboratory results (Median - IQR)** |  |  |  |
| White Blood Cells | 7,460 (5,770 – 11,220) | - | - |
| Neutrophils | 5,980 (4,075 – 8,760) | - | - |
| Lymphocytes | 1,000 (745 – 1,370) | - | - |
| Platelets | 230,000 (202,000 – 270,000) | - | - |
| C reactive protein (mg/L) | 103.18 (64.51 – 189.66) | - | - |
| Erythrocyte sedimentation rate (mm/hr) | n=34/35, 30.5 (17.3 – 37.5) | - | - |
| Albumin (g/dL) | n=34/35, 3.5 (3.35 – 3.77) | - | - |
| Total bilirubin (μmol/L) | 0.55 (0.33 – 0.66) | - | - |
| Glutamic oxaloacetic transaminase (U/L) | 40 (29.5 – 68.0) | - | - |
| Glutamic pyruvic transaminase (U/L) | 46 (34.0 – 82.5) | - | - |
| Prothrombin time (seconds) | n=34/35, 10.4 (10.2 – 10.6) | - | - |
| INR | n=34/35, 0.985 (0.97 – 1.00) | - | - |
| Urea (mg/dL) | 14.7 (11.4 – 19.0) | - | - |
| Creatinine (mg/dL) | 0.78 (0.68 – 0.91) | - | - |
| Creatine kinase (U/L) | 78 (52 – 168) | - | - |
| D dimer (mg/L) | 0.51 (0.31 – 0.92) | - | - |
| Ferritin (ng/mL) | n=34/35, 1118.5 (781.9 – 1834.8) | - | - |
| Lactate dehydrogenase (U/L) | 298 (270 – 353.5) | - | - |
| Procalcitonin (ng/mL) | 0.122 (0.085 – 0.294) | - | - |
| Troponin T (ng/mL) | 0.006 (0.004 – 0.007) | - | - |
| **Post-COVID time (days, Median – IQR)** | 217 (186 – 238) | - | - |
| **Zung score (Median -IQR)** | 32 (28.5 – 38.5) | - | - |
| **COMPASS 31 score (Median -IQR)** | 9 (4.5 – 14.5) | - | - |
| **Constitutional Symptoms** | 10 (28.6%) | - | - |
| Anorexia | 4 (11.4%) | - | - |
| Chills | 7 (20.0%) | - | - |
| Weight loss | 6 (17.1%) | - | - |
| **Dermatological symptoms** | 14 (40.0%) | - | - |
| Exanthema | 11 (31.4%) | - | - |
| Blisters | 3 (8.6%) | - | - |
| Skin sensitivity | 4 (11.4%) | - | - |
| **Musculoskeletal symptoms** | 26 (74.3%) | - | - |
| Sarcopenia | 10 (28.6%) | - | - |
| Weakness | 14 (40.0%) | - | - |
| Myalgia | 12 (34.3%) | - | - |
| Back pain | 20 (57.1%) | - | - |
| Arms/legs heaviness | 19 (54.3%) | - | - |
| Inability to walk | 5 (14.3%) | - | - |
| Body pain | 13 (37.1%) | - | - |
| Arthralgia | 26 (74.3%) | - | - |
| **Cardiac symptoms** | 14 (40.0%) | - | - |
| Palpitations | 9 (25.7%) | - | - |
| Chest tightness | 12 (34.3%) | - | - |
| Tachycardia | 9 (25.7%) | - | - |
| Edema | 5 (14.3%) | - | - |
| **Pulmonary symptoms** | 16 (45.7%) | - | - |
| Chest pain | 9 (25.7%) | - | - |
| Dyspnea | 9 (25.7%) | - | - |
| Wet cough | 3 (8.6%) | - | - |
| Dry cough | 7 (20.0%) | - | - |
| Pleurisy | 5 (14.3%) | - | - |
| Rhinorrhea | 9 (25.7%) | - | - |
| **Gastrointestinal symptoms** | 11 (31.4%) | - | - |
| Sickness | 7 (20.0%) | - | - |
| Abdominal pain | 9 (25.7%) | - | - |
| **Neurological symptoms** | 19 (54.3%) | - | - |
| Paresthesia | 12 (34.3%) | - | - |
| Attention disorders | 8 (22.9%) | - | - |
| Memory disorders | 9 (25.7%) | - | - |
| Headache | 14 (40.0%) | - | - |
| Dizziness | 10 (28.6%) | - | - |
| **Sensory and glandular symptoms** | 15 (42.9%) | - | - |
| Xerophthalmia | 9 (25.7%) | - | - |
| Xerostomia | 8 (22.9%) | - | - |
| Tooth Loss | 1 (2.9%) | - | - |
| Anosmia | 7 (20.0%) | - | - |
| Ageusia | 2 (5.7%) | - | - |
| Vision disorders | 7 (20.0%) | - | - |
| Eye pain | 13 (37.1%) | - | - |
| Pharyngitis | 8 (22.9%) | - | - |

ACE: Angiotensin-converting enzyme; ARB II: Angiotensin receptor blockers 2; BMI: Body mass index; ICU: Intensive care unit; IQR: Interquartile range; INR: International normalized ratio; PCS: post-COVID syndrome; PPC: Pre-pandemic healthy control; SD: Standard deviation; SOFA: Sequential organ failure assessment.
