# Supplementary material for: NET Biomarkers in COVID-19 and Post-COVID Syndrome: a Comprehensive Analysis
Source: J Clin Immunol. 2026 Feb 2;46(1):19. doi: 10.1007/s10875-026-01980-9 (PMC12909450; doi:10.1007/s10875-026-01980-9)
Supplement: Supplementary file 2 — Supplementary file2 (DOCX 17 KB) [file 10875_2026_1980_MOESM2_ESM.docx]

**Table S2.** Correlation of NET-associated biomarkers with clinical and inflammatory markers in acute COVID-19 patients.

| **x** | **y** | **r** | **P Values** |
| --- | --- | --- | --- |
| IgG Anti-SARS-CoV-2 | MPO-DNA complex | -0.3609869 | 0.0175568603 |
| CRP | MPO-DNA complex | 0.3122191 | 0.0079045248 |
| GM-CSF levels | MPO-DNA complex | 0.7078795 | 0.0127995981 |
| IL-8 levels | MPO-DNA complex | 0.1913262 | 0.0182165237 |
| Lymphocyte count | MPO-DNA complex | -0.1577984 | 0.0346355100 |
| Neutrophils count | MPO-DNA complex | 0.4995830 | 0.0047216309 |
| WBC count | MPO-DNA complex | 0.4471029 | 0.0480066705 |
| Admission age | Elastase-DNA complex | -0.3578719 | 0.0445336408 |
| IgM anti-β2GP1 Abs | Elastase-DNA complex | -0.5484381 | 0.0298837832 |
| CPR | Elastase-DNA complex | 0.3082479 | 0.0475643389 |
| IFN-γ levels | Elastase-DNA complex | -0.5796397 | 0.0466957094 |
| IL-4 levels | Elastase-DNA complex | -0.5203586 | 0.0450085992 |
| IL-8 levels | Elastase-DNA complex | 0.2460255 | 0.0199033653 |
| Lymphocyte count | Elastase-DNA complex | -0.3106086 | 0.0401493860 |
| Neutrophil count | Elastase-DNA complex | 0.5128497 | 0.0006468210 |
| WBC count | Elastase-DNA complex | 0.4722184 | 0.0089572834 |
| ALT | MPO | 0.4711066 | 0.0135787822 |
| IgM anti-β2GP1 Abs | MPO | -0.7568313 | 0.0039561923 |
| CRP | MPO | 0.5531725 | 0.0038562324 |
| Ferritin | MPO | 0.4285186 | 0.0392406694 |
| IL-10 levels | MPO | 0.3702913 | 0.0259841339 |
| IL-8 levels | MPO | 0.4284551 | 0.0008060693 |
| Neutrophil count | MPO | 0.6793754 | 0.0002074801 |
| PaO2-FiO2 | MPO | -0.5284242 | 0.0044620320 |
| WBC count | MPO | 0.6492562 | 0.0014475204 |

x and y represent the variables tested for correlation; r indicates Spearman’s correlation coefficient; p values represent the level of statistical significance. **Abbreviations:** Abs, antibodies; ALT, alanine transaminase; β2GP1, β2 glycoprotein 1; CRP, C-reactive protein; GM-CSF, granulocyte-macrophage colony-stimulating factor; IFN, interferon; IL, interleukin; MPO, myeloperoxidase; PaO₂-FiO₂, arterial oxygen partial pressure to fractional inspired oxygen ratio; WBC, white blood cell.
