# Supplementary material for: NET Biomarkers in COVID-19 and Post-COVID Syndrome: a Comprehensive Analysis
Source: J Clin Immunol. 2026 Feb 2;46(1):19. doi: 10.1007/s10875-026-01980-9 (PMC12909450; doi:10.1007/s10875-026-01980-9)
Supplement: Supplementary file 3 — Supplementary file3 (DOCX 17 KB) [file 10875_2026_1980_MOESM3_ESM.docx]

**Table S3.** Correlation of NET-associated biomarkers with inflammatory markers in PCS patients.

| **x** | **y** | **r** | **P Values** |
| --- | --- | --- | --- |
| Aldolase C Abs | MPO-DNA complex | 0.3742169896 | 0.0309179805 |
| GM-CSF Abs | MPO-DNA complex | 0.3977064526 | 0.0467360907 |
| HSPG Abs | MPO-DNA complex | 0.4017944686 | 0.0429950118 |
| IFN-α2 Abs | MPO-DNA complex | 0.3828613041 | 0.0071837461 |
| IFN-αF Abs | MPO-DNA complex | 0.2161585996 | 0.0407820703 |
| IFN-λ3 Abs | MPO-DNA complex | 0.4463923632 | 0.0416150684 |
| IL-1β Abs | MPO-DNA complex | 0.3981612427 | 0.0037653835 |
| IL-6 levels | MPO-DNA complex | 0.3743336362 | 0.0252957785 |
| BMI | MPO-DNA complex | -0.3191838743 | 0.0060417183 |
| Vimentin Abs | MPO-DNA complex | 0.3993551733 | 0.0211063133 |
| IgM ACA | MPO | -0.4716186910 | 0.0347408310 |
| BPI Abs | MPO | -0.2243236202 | 0.0403956135 |
| CD8B Abs | MPO | -0.3069138721 | 0.0166836382 |
| HCoV-HKU1 S Abs | MPO | -0.4038558374 | 0.0147891722 |
| IFN-λ1 Abs | MPO | -0.2873721385 | 0.0097092649 |
| IL-6 levels | MPO | 0.6349703074 | 0.0325661934 |
| ACE2 Abs | Elastase-DNA complex | 0.3018384999 | 0.0310945995 |
| IgM anti-β2GP1 Abs | Elastase-DNA complex | 0.0005057493 | 0.0116965673 |
| CD8B Abs | Elastase-DNA complex | -0.3321204576 | 0.0115993025 |
| Collagen VI Abs | Elastase-DNA complex | -0.2699810382 | 0.0230754664 |
| IL-5 levels | Elastase-DNA complex | 0.2404427583 | 0.0288944428 |

x and y represent the variables tested for correlation; r indicates Spearman’s correlation coefficient; p values represent the level of statistical significance. **Abbreviations:** Abs, antibodies; ACA, anticardiolipin antibodies; ACE2, angiotensin-converting enzyme 2; β2GP1, β2 glycoprotein 1; BMI, body mass index; BPI, bactericidal/permeability-increasing protein; CD8B, CD8 beta chain; GM-CSF, granulocyte-macrophage colony-stimulating factor; HCoV-HKU1 S, human coronavirus HKU1 spike protein; HSPG, heparan sulfate proteoglycan; IFN, interferon; IL, interleukin; MPO, myeloperoxidase; TNF, tumor necrosis factor; TTG, tissue transglutaminase.
