# Supplementary material for: NET Biomarkers in COVID-19 and Post-COVID Syndrome: a Comprehensive Analysis
Source: J Clin Immunol. 2026 Feb 2;46(1):19. doi: 10.1007/s10875-026-01980-9 (PMC12909450; doi:10.1007/s10875-026-01980-9)
Supplement: Supplementary file 4 — Supplementary file4 (DOCX 20 KB) [file 10875_2026_1980_MOESM4_ESM.docx]

**Supplementary Methods – NET Biomarkers in COVID-19 and Post-COVID Syndrome**

**Human neutrophil purification**

Whole blood samples were collected from healthy volunteer donors into EDTA tubes by an experienced phlebotomist. Leukocytes were isolated through red blood cell (RBC) sedimentation by mixing whole blood with an equal volume of 6% (w/v) dextran (02101514-CF, MP Biomedicals) diluted in RPMI-1640 (with L-glutamine, without calcium and magnesium; 11875093, Gibco). The mixture was incubated at room temperature (RT) for 40 minutes. The upper phase, enriched in leukocytes and poor in RBCs, was carefully pipetted on top of Ficoll-Hypaque 1.077 g/mL (10771, Sigma-Aldrich) at a 2:1 volume ratio and centrifuged at 600 × g for 20 minutes at RT without braking. After centrifugation, the top three layers—diluted plasma, peripheral blood mononuclear cells (PBMCs), and Ficoll—were gently removed, leaving a red pellet containing RBCs and polymorphonuclear cells (PMNs). This pellet was resuspended in ACK (ammonium-chloride-potassium) lysing buffer and incubated for 15 minutes at RT to lyse residual RBCs. The sample was then centrifuged at 1500 rpm for 5 minutes at 4°C, and the supernatant was carefully aspirated. PMNs were resuspended in complete medium (RPMI-1640 with 10% heat-inactivated fetal bovine serum (FBS, s18b, Biowest), 100 U/mL penicillin, and 100 µg/mL streptomycin (p4333, Sigma-Aldrich) and 25mM HEPES (15630-080, Gibco). An aliquot was taken to assess neutrophil count and viability using the trypan blue exclusion assay. Neutrophil preparations were confirmed to have a purity of at least 95%, based on nuclear morphology.

***In-House* Control NETs**

To minimize inter-assay variability and address the absence of an international standard for measuring NET complexes, a stock solution of NET standard, defined as 100% NET, was prepared and used to generate a standard curve as described for Guy et al. [1]. For this purpose, neutrophils were isolated from five healthy volunteer donors. The purified neutrophils were resuspended in complete RPMI-1640 medium and seeded at a density of 2.5 × 10⁵ cells per well in a 24-well culture plate (92024, TPP). The cells were then stimulated with 100 nM PMA (P8139, Sigma-Aldrich) as a positive control or left unstimulated as a negative control for 4 hours in an atmosphere of 5% CO₂ and 95% air. After incubation, the supernatants were carefully collected from the wells and transferred to sterile tubes. The remaining cells were treated with 10 U/mL DNase (79254, Qiagen) diluted in RPMI-1640 to disrupt the NETs. Following a 30-minute incubation at 37°C, the plates were centrifuged at 1500 rpm for 5 minutes, and the supernatants were collected. Finally, the supernatants from stimulated and unstimulated neutrophils, obtained from five independent donors, were pooled for subsequent analysis by ELISA.

**Detection of MPO-DNA complexes**

To determine the presence of MPO-DNA complexes in serum, we adapted a sandwich ELISA method previously described by Kessenbrock et al. [2] and Zuo et al. [3]. High-binding 96-well plates (Greiner Bio-One, 762071) were coated overnight at 4°C with 100 μl per well of anti-MPO monoclonal antibody (Bio-Rad 0400-0002), diluted to a concentration of 1 μg/mL in PBS 1X (70011044, Life technologies). The next day, the antibody solution was removed and the plates were washed three times using 200 µL per well of wash buffer (0,05% Tween20 (P1379, Sigma-Aldrich) in PBS 1X). Then, the plates were blocked with 200 µL of 5% bovine serum albumin (BSA-A3912, Sigma-Aldrich) + 0,05% Tween20 in PBS 1X for 2 hours at RT with agitation at 30 rpm. The plates were subsequently washed five times with wash buffer and then incubated with 100 µL of control samples prepared in a series of consecutive 1:2 dilutions, starting from 1:5 dilution (NETs from the supernatants of neutrophils, either non-stimulated or stimulated with PMA) or 10% serum sample. The samples were diluted in 5% BSA in PBS 1X and incubated at RT with agitation at 30 rpm for 90 minutes. After washing the plates five times with wash buffer, 100 µL per well of peroxidase-conjugated anti-DNA antibody (11774425001, Roche-Cell Death Detection ELISAPLUS Anti-DNA POD) diluted 1:100 in 5% BSA + 0.05% Tween20 in PBS 1X was added and incubated at RT with agitation at 30 rpm for 90 minutes. After five more washes, 100 µL per well of 3,3’,5,5’-tetramethylbenzidine TMB-substrate (T2885, Sigma-Aldrich) was used for ELISA development. The plates were incubated at RT in the dark for 5 minutes and the reaction was stopped by adding 100 µL per well of 2N stop solution (H_2_SO_4_). The absorbance was measured at a wavelength of 450 nm with a reference correction wavelength at 655 nm using a microplate absorbance reader (iMark, BIO-RAD). Absorbances from *in vitro*-prepared NETs controls included on every plate, were used for establish a standard curve plotting the optical density of the dilutions against % NET-standard content, based on the methodology used by Guy et al [1]. The levels of MPO-DNA complexes in serum samples were calculated using the NET-standard curve, and the results were reported as a percentage relative to the "NET-standard”.

**Detection of elastase-DNA complexes**

To detect elastase-DNA complexes in serum, we adapted a sandwich ELISA based on the PMN Elastase Human ELISA Kit (ab119553, Abcam), following the manufacturer’s instructions with a modification in the conjugate used for complex detection. Briefly, 96-well plates coated with a polyclonal antibody against PMN elastase were incubated with 100 µL of control samples (NETs derived from the supernatants of neutrophils, either unstimulated or stimulated with PMA) or serum samples diluted 1:10. After a 1-hour incubation at RT with agitation at 30 rpm, the wells were washed four times as specified in the kit protocol. Next, 100 µL of DNA-POD conjugated antibody, diluted 1:100 (from the Roche Cell Death Detection ELISAPLUS kit) was added and incubated for 1 hour at RT with agitation at 30 rpm. Following four additional washes, TMB substrate solution was added for color development and incubated for 20 minutes. The reaction was stopped by adding stop solution to each well. Absorbance was measured at 450 nm with a reference wavelength correction at 655 nm.

**NETosis induction by serum samples**

Neutrophils were isolated from freshly collected blood samples from healthy volunteer donors, following the human neutrophil purification protocol described above. A total of 2.5 × 10⁵ neutrophils per well were seeded onto coverslips coated with 0.01% (w/v) poly-L-lysine (P8920, Sigma-Aldrich) in a 24-well culture plate. Cells were either left unstimulated, stimulated with 100 nM PMA (controls), or cultured with 10% serum. The plates were incubated at 37°C in an atmosphere of 5% CO₂ and 95% air. Each condition was performed in duplicate wells. After 4 hours of incubation, the cells were fixed with 4% paraformaldehyde (150146, MP Biomedicals) for 15 minutes at RT. Following two washes with 1× PBS, the fixed cells were permeabilized for 5 minutes with 0.5% Triton X-100 (G5516, Sigma-Aldrich) and subsequently blocked with 5% bovine serum albumin (A3912, Sigma-Aldrich) in PBS for 30 minutes at RT. To visualize NETs, primary antibody against myeloperoxidase (MPO; Ab9535, Abcam) was applied at a 1:30 dilution in blocking solution and incubated overnight at 4°C. After three washes with 1× PBS containing 0.2% Triton X-100, a sequential incubation was performed using a 1:100 dilution of anti-DNA/Histone H1 antibody (Mab3864, Sigma-Aldrich) for 2 hours at RT. Following three additional washes, primary antibodies were detected with Alexa Fluor 488-conjugated (A11001, Thermo Fisher Scientific) and Alexa Fluor 546-conjugated (A11010, Thermo Fisher Scientific) secondary antibodies, each diluted 1:1000 in blocking solution. After another series of three washes, DNA was stained with Hoechst 33342 (H3570, Thermo Fisher Scientific), and coverslips were mounted using 70% glycerol. Images were acquired using a Leica DMi8 microscope and processed with Leica Application Suite X (LAS-X) software.

**References**

1. Guy A, Favre S, Labrouche-Colomer S, Deloison L, Gourdou-Latyszenok V, Renault M-A, et al. High circulating levels of MPO-DNA are associated with thrombosis in patients with MPN. Leukemia. England; 2019. p. 2544–8.

2. Kessenbrock K, Krumbholz M, Schönermarck U, Back W, Gross WL, Werb Z, et al. Netting neutrophils in autoimmune small-vessel vasculitis. Nat Med. 2009;15:623–5.

3. Zuo Y, Yalavarthi S, Shi H, Gockman K, Zuo M, Madison JA, et al. Neutrophil extracellular traps in COVID-19. JCI Insight. 2020;
